# Constitutively active follicle-stimulating hormone receptor enables androgen-independent spermatogenesis

**DOI:** 10.1172/JCI96794

**Published:** 2018-03-26

**Authors:** Olayiwola O. Oduwole, Hellevi Peltoketo, Ariel Poliandri, Laura Vengadabady, Marcin Chrusciel, Milena Doroszko, Luna Samanta, Laura Owen, Brian Keevil, Nafis A. Rahman, Ilpo T. Huhtaniemi

**Affiliations:** 1 Institute of Reproductive and Developmental Biology (IRDB), Department of Surgery and Cancer, Imperial College London, Hammersmith Hospital Campus, London, United Kingdom.; 2 Laboratory of Cancer Genetics and Tumor Biology, Cancer and Translational Medicine Research Unit/Laboratory Medicine, Biocenter Oulu and Faculty of Medicine, University of Oulu, Oulu, Finland.; 3 Department of Molecular and Clinical Sciences, St. George’s University of London, London, United Kingdom.; 4 Department of Target Sciences, GlaxoSmithKline, London, United Kingdom.; 5 Department of Physiology, University of Turku, Turku, Finland.; 6 Department of Zoology, School of Life Sciences, Ravenshaw University, Cuttack, India.; 7 Biochemistry Department, University Hospital of South Manchester, Manchester, United Kingdom.; 8 Department of Reproduction and Gynecological Endocrinology, Medical University of Bialystok, Bialystok, Poland.

**Keywords:** Endocrinology, Reproductive Biology, Fertility, Reproductive biochemistry

## Abstract

Spermatogenesis is regulated by the 2 pituitary gonadotropins, luteinizing hormone (LH) and follicle-stimulating hormone (FSH). This process is considered impossible without the absolute requirement of LH-stimulated testicular testosterone (T) production. The role of FSH remains unclear because men and mice with inactivating FSH receptor (FSHR) mutations are fertile. We revisited the role of FSH in spermatogenesis using transgenic mice expressing a constitutively strongly active FSHR mutant in a LH receptor–null (*LHR*-null) background. The mutant FSHR reversed the azoospermia and partially restored fertility of *Lhr*^–/–^ mice. The finding was initially ascribed to the residual Leydig cell T production. However, when T action was completely blocked with the potent antiandrogen flutamide, spermatogenesis persisted. Hence, completely T-independent spermatogenesis is possible through strong FSHR activation, and the dogma of T being a sine qua non for spermatogenesis may need modification. The mechanism for the finding appeared to be that FSHR activation maintained the expression of Sertoli cell genes considered androgen dependent. The translational message of our findings is the possibility of developing a new strategy of high-dose FSH treatment for spermatogenic failure. Our findings also provide an explanation of molecular pathogenesis for Pasqualini syndrome (fertile eunuchs; LH/T deficiency with persistent spermatogenesis) and explain how the hormonal regulation of spermatogenesis has shifted from FSH to T dominance during evolution.

## Introduction

It is textbook knowledge that spermatogenesis is impossible without luteinizing hormone–stimulated (LH-stimulated) testicular testosterone (T) production (1, 2). Human mutations and genetically modified mice provide further proof for this, as men harboring inactivating *LHB* and *LHCGR* mutations are hypogonadal and azoospermic, with knockout mice for the same genes exhibiting a similar phenotype (2). Surprisingly, the role of follicle-stimulating hormone (FSH), the other endocrine stimulus of spermatogenesis, has remained elusive. Although a long-held notion ascribes the pubertal initiation and maintenance of normal spermatogenesis to FSH (1), phenotypes of inactivating *FSHB* (3) and FSH receptor (*FSHR*) (4) mutations in men and knockout mice for the cognate genes (5, 6) prompt a different conclusion. With the exception of men with *FSHB* mutations (3), all others preserve spermatogenesis and fertility. Hence, the current view is that FSH improves spermatogenesis qualitatively and quantitatively in additive fashion with T, but is not necessary for male fertility per se. While T can maintain spermatogenesis without FSH, the reverse has never been demonstrated (7). Despite the crucial role of T, the specific phase of spermatogenesis that is absolutely androgen dependent and the distinct functions of T and FSH have not been completely delineated. Because of their entirely different mechanisms of action, FSH acting through a GPCR with cAMP as second messenger and T activating the androgen receptor (AR), a nuclear transcription factor, it is justified to hypothesize that each hormone has distinct and irreplaceable effects.

To delineate the cryptic role of FSH in spermatogenesis, we used the transgenic mouse model expressing a constitutively strongly activating *Fshr* point mutation (*Fshr*-CAM; *D580H)* in Sertoli cells (SC) under the human anti-Müllerian hormone promoter (8). These mice were crossed with female heterozygous *Lhr^–/–^* mice (9) with the expectation that the role of FSH could in this way be amplified and studied in isolation from the LH/T effects. The unexpected findings during the experiments challenge the dogma of T dependence of spermatogenesis because missing T action was completely compensated for by strong FSH stimulation. Our findings therefore herald potentially novel strategies into the treatment of human spermatogenic failure and suggest mechanisms for some unexplained aberrations of human spermatogenesis and the evolution of its hormonal regulation.

## Results and Discussion

**Figure 1:**
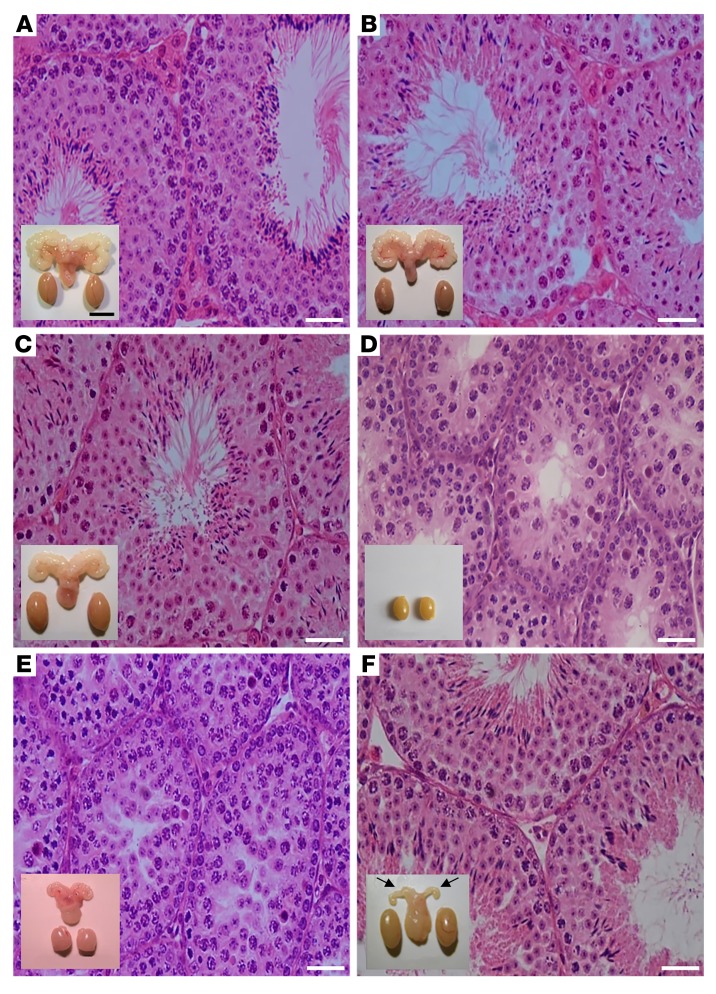
Testicular histology and macroscopic views of testes and urogenital blocks from different mouse genotypes and from flutamide-treated animals. Representative views of (**A**) WT, (**B**) *Fshr*-CAM, (**C**) *Fshr*-CAM/*Lhr^–/–^*, and (**D**) *Lhr^–/–^* mice (*n* = 5–8/group). **A**–**C** show normal spermatogenesis and testis and SV sizes. In **D**, spermatogenesis is shown as arrested at the RS stage, with small testes and rudimentary SV (not shown). (**E**) Treatment of WT mice (*n* = 5/group) with antiandrogen flutamide arrested spermatogenesis at RS stage, with reduced testis and SV sizes. (**F**) Identical treatment of *Fshr*-CAM/*Lhr^–/–^* mice (*n* = 5/group) had no apparent effect on spermatogenesis and testis size, but reduced SV sizes (arrows in **F**). Scale bars: 50 μm; 10 mm (insets).

Our transgenic mouse model expressed a constitutively strongly activating (cAMP response >10-fold above basal) *Fshr* point mutation (*Fshr*-CAM; *D580H)* under the human anti-Müllerian hormone promoter (8), providing strict SC-specific expression of the transgene (10) and almost a 20-fold *Fshr* expression at mRNA level in comparison with WT mice. Unlike females with a robust ovarian and reproductive phenotype (8), the male littermates had no apparent abnormalities. Their testicular architecture (Figure 1B) and hormonal parameters (Supplemental Table 1; supplemental material available online with this article; https://doi.org/10.1172/JCI96794DS1) were as in WT (Figure 1A and Supplemental Table 1). Immunohistochemical localization of the FSH protein and RNAscope in situ hybridization of *Fshr* mRNA were confined to SC (Supplemental Figure 1), indicating that functionally meaningful leakage of the *Fshr*-CAM transgene to Leydig cells (LC) is highly unlikely. In the absence of any phenotype of the *Fshr*-CAM males, it is apparent that physiological FSH concentrations provide maximal SC stimulation. This may explain why only 2 activating *FSHR* mutations, detected serendipitously, have been described. One was a hypophysectomized man with persistent spermatogenesis (11) and the other a man with normal spermatogenesis in the absence of circulating FSH (12). Furthermore, testicular function appears normal in men with pituitary adenomas secreting excessive FSH (13, 14). Hence, there may be no phenotypic effect of enhanced FSH action in otherwise healthy men.

Strong Fshr activation in the double-mutant *Fshr*-CAM/*Lhr*^–/–^ mice surprisingly reversed the hypogonadism and infertility of the *Lhr^–/–^* mice, with development of a near-normal male phenotype with increased testicular size and spermatogenesis (Figure 1C), comparable to that in WT and *Fshr*-CAM littermates (Figure 1, A and B). These mice, however, presented with delayed puberty (Supplemental Figure 2A). The anogenital distance, another androgen-dependent developmental parameter, was similar in the *Fshr*-CAM/*Lhr^–/–^* mice and control littermates at weaning (P20), but at puberty and thereafter, it was approximately 10% and 40% shorter in the *Fshr*-CAM/*Lhr^–/–^* and *Lhr*^–/–^ mice, respectively (Supplemental Figure 2B). Mating tests showed similar mounting behavior in *Fshr*-CAM/*Lhr^–/–^* mice and WT littermates, with evidence for copulatory plugs in females. However, the *Fshr*-CAM/*Lhr^–/–^* breeding pairs had lower frequencies of pregnancies and smaller litter sizes (Supplemental Table 2), with no evidence for embryonic lethality in offspring.

**Figure 2:**
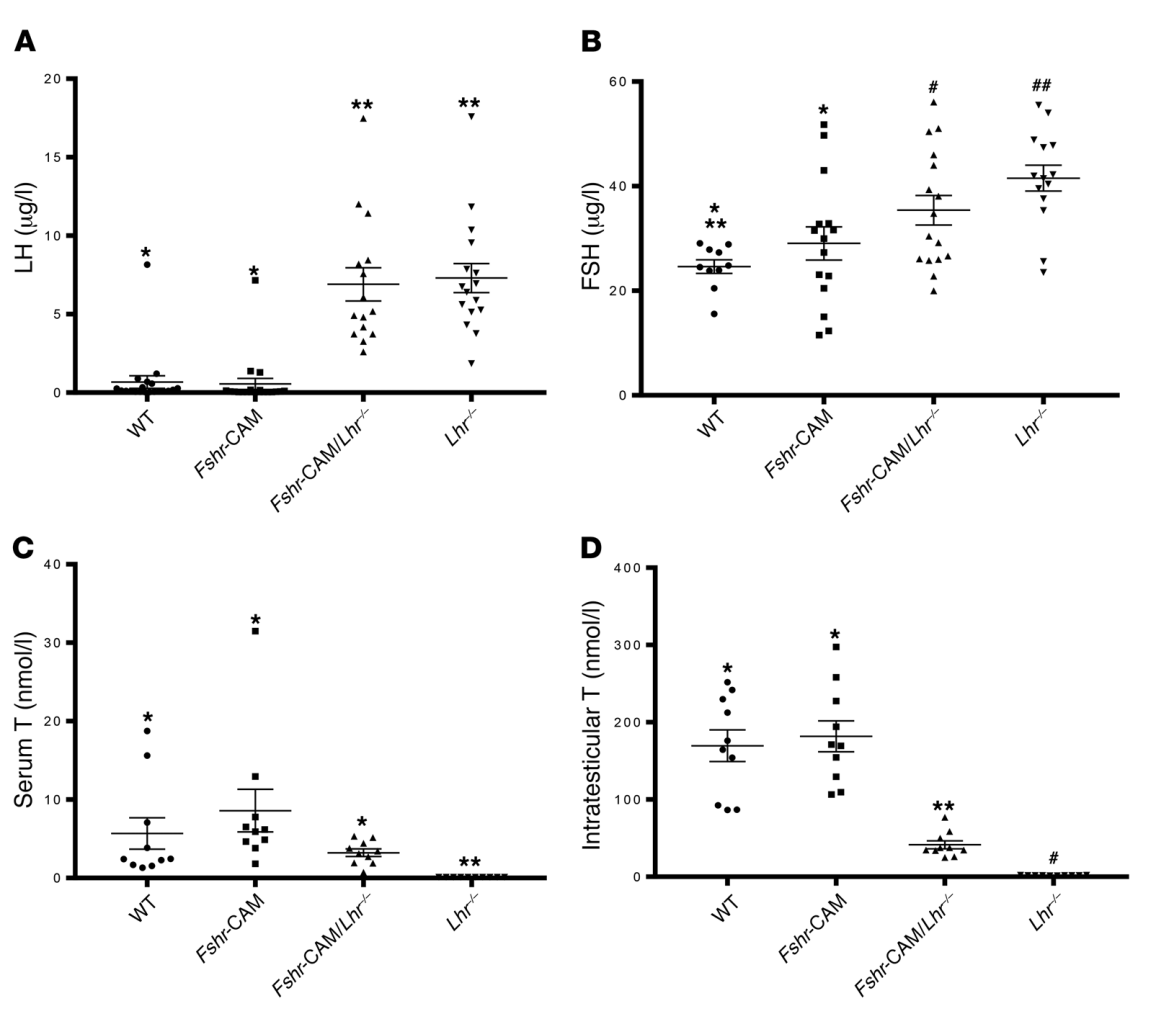
Hormone analyses. (**A**) Serum LH, (**B**) serum FSH, (**C**) serum T, and (**D**) iTT. Data represent mean ± SEM. *n* = 10–15 individual samples/group. Groups with different symbols differ significantly from each other (*P* < 0.05; ANOVA/Newman-Keuls).

LH concentrations were markedly (10-fold) and FSH marginally (1.5-fold) elevated in the mice with *Lhr^–/–^* genotypes (Figure 2, A and B). Serum T in the *Lhr*^–/–^ mice was very low (<0.01 nmol/l), while in *Fshr*-CAM/*Lhr^–/–^* mice, it recovered to about 40% of WT levels (Figure 2C). Similar trends, though with greater differences between genotypes, were observed in intratesticular T (iTT) concentrations (Figure 2D). The increased seminal vesicle (SV), epididymis, and testis weights (Supplemental Figure 2, C–E) and the observed testicular descent in *Fshr*-CAM/*Lhr^–/–^* mice demonstrated biological action of the partially recovered T production. However, the lack of LH suppression indicated that the recovered T production was insufficient to evoke negative feedback.

Sizes of the double-mutant mouse testes were indistinguishable from those of WT (Table 1 and Supplemental Figure 2E) and reflected on the appearance of fully developed seminiferous tubules, with typical stages of the seminiferous epithelial cycle and presence of elongated and mature spermatids (Figure 1C). A noticeable difference common to both *Lhr^–/–^* genotypes was the apparent lack of mature LC (Figure 1, C and D). Stereological assessment per testis (Table 1) and per mg testis (Supplemental Table 3) indicated approximately 15% less SC per testis of the *Fshr*-CAM/*Lhr*^–/–^ mice, with no difference in tubular diameter or the number of spermatogonia A and B, when compared with WT. The round spermatid (RS) number and RS/SC and spermatogonia/SC ratios were also similar; however, the number of elongated spermatids per testis was about half that in WT. This observation was different in the *Lhr*^–/–^ testes, where tubular diameter was approximately half of WT, the number of spermatogonia A was doubled, RS and RS/SC ratios were drastically reduced, the spermatogonia/SC ratio was increased, and elongated spermatids were completely absent (Figure 1D).

**Figure 3:**
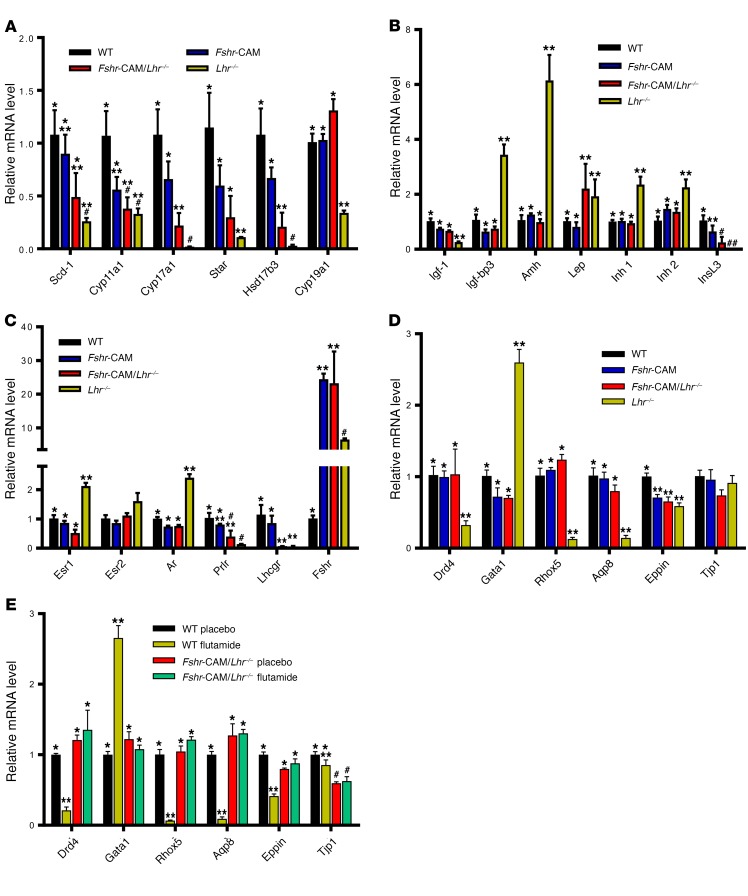
Relative mRNA expression in testes. (**A**) Steroidogenic genes. (**B**) Hormones and growth factors. (**C**) Hormone receptors. (**D**) Androgen-regulated (*Drd5*, *Rhox5*, *Eppin*, and *Tjp1*), postmeiotic germ cell–specific (*Aqp8*), and germ cell–regulated (*Gata1*) genes in WT, *Fshr*-CAM, *Fshr*-CAM/*Lhr*^–/–^, and *Lhr*^–/–^ testes. In contrast with the LC genes downregulated in *Lhr*^–/–^ testes (**A**), we identified several upregulated SC-specific genes (**B** and **C**). Expression of 3 steroid receptor genes with mixed localization, namely, *Esr1*, *Esr2*, and *Ar*, also resembled that of the SC-specific genes (**C**). The increased proportion of SC per unit weight in *Lhr*^–/–^ testes (Table 1 and Supplemental Table 3) apparently explains, at least partly, the enrichment of the SC genes. Expression of these genes became normalized in the *Fshr*-CAM/*Lhr*^–/–^ mice, in accordance with the normalization of testis size and proportions of the different cell types. (**E**) Effect of flutamide treatment on expression of androgen-regulated genes in WT and *Fshr*-CAM/*Lhr*^–/–^ mice. Data represent mean ± SEM. *n* = 3 samples/group. Bars with different symbols differ significantly from each other (*P* < 0.05; ANOVA/Newman-Keuls).

At this stage, we hypothesized that the recovery of spermatogenesis in the *Fshr*-CAM/*Lhr^–/–^* mice was due to the partially recovered T production by rudimentary LC, stimulated by the well-documented FSH-responsive paracrine signaling from SC (2, 15–17). Accordingly, the expression of LC-specific genes *Scd1*, *Star*, *Cyp11a1*, *Cyp17a1*, and *Hsd17b3* was slightly increased in the double-mutant mice over *Lhr^–/–^* mice (Figure 3A), while the expression levels of SC genes roughly paralleled the proportion of SC in the testes (Figure 3, B and C). Immunohistochemistry of StAR, HSD17B3, CYP17A1, and HSD3B1 demonstrated that the steroidogenic transport protein and enzymes were solely confined to LC in all genotypes (Supplemental Figure 3), excluding ectopic SC T production. The observed iTT concentration of approximately 40 nmol/l in the *Fshr*-CAM/*Lhr*^–/–^ mice is in fact sufficient to initiate spermatogenesis in T-treated *Lhr*^–/–^ mice (18).

We next addressed the role of the residual T levels in the unexpected activation of spermatogenesis in the *Fshr*-CAM/*Lhr^–/–^* mice by eliminating the effect of this androgen through treatment with the potent antiandrogen flutamide. As anticipated, in WT control mice, flutamide induced shrinkage of SVs and testes along with complete cessation of spermatogenesis at the RS stage (Figure 1E). Stereological assessment of the testes after treatment (Supplemental Table 4) confirmed the efficacy of flutamide treatment. Previously, the same antiandrogen treatment in 12-month-old *Lhr^–/–^* mice completely blocked spermatogenesis at the RS stage; however, spermatogenesis was qualitatively complete in control *Lhr^–/–^* mice and driven by the residual low iTT level (2% of normal) (19). Surprisingly, identical antiandrogen treatment of *Fshr*-CAM/*Lhr^–/–^* mice brought about only shrinkage of the SVs, without affecting testicular size, cell-type composition, and sperm maturation (Figure 1F and Supplemental Table 4). Hence, upon complete blockage of androgen action, the constitutively active FSHR maintained spermatogenesis in these mice.

Previously, a study on the role of FSH in mouse spermatogenesis compared *Lhr*^–/–^ and hypogonadal gonadotropin–deficient *hpg* mice, expressing through transgenesis either human *FSH* or a mildly constitutively active form of human *FSHR* (20). Both presented with normal to high-normal FSH action and low T levels, which, relative to *hpg* controls, led to increased SC numbers, enhanced spermatogonial proliferation, and some meiotic development, but no mature spermatids. FSH stimulation alone in these models was unable to evoke complete spermatogenesis without the critical involvement of LH-stimulated T production. In contrast, our findings demonstrate that stimulation of spermatogenesis with strong FSH effect alone is possible.

Quantification of selected androgen-dependent SC genes *Drd4,* *Rhox 5,* and *Eppin* (2) demonstrated their clearly decreased expression in the flutamide-treated WT testes in agreement with their low expression in the androgen-deprived *Lhr^–/–^* testes (Figure 3, D and E). In contrast, no reduction in the high expression of these androgen-dependent genes was found in flutamide-treated *Fshr*-CAM/*Lhr^–/–^* testes (Figure 3E), indicating that the strong *Fshr*-CAM signaling was able to maintain the expression of genes considered strictly androgen regulated. A similar expression pattern was found with the indirectly androgen-dependent postmeiotic germ cell–specific gene *Aqp8* (21), apparently reflecting the persistence of testicular postmeiotic germ cells, normally present in the testis only through androgen action, but maintained by Fshr activation in the flutamide-treated *Fshr*-CAM/*Lhr^–/–^* testes. Another SC-specific gene, *Gata-1*, is resistant to androgen action, but downregulated by paracrine effects from postmeiotic germ cells (22). Hence, it was upregulated in the flutamide-treated WT testes, consequent to postmeiotic germ cell depletion, but not in the *Fshr*-CAM/*Lhr^–/–^* testes with full spermatogenesis. Expression of *Tjp1*, a loosely androgen-regulated SC gene (23), was largely unaffected by the genetic and hormonal manipulations. Hence, *Fshr-*CAM expression could substitute for missing androgen action in the maintenance of testicular androgen–dependent gene expression, providing a mechanism for the unexpected androgen-independent spermatogenesis.

T and FSH are assumed to regulate spermatogenesis through distinct and nonoverlapping signaling mechanisms (24, 25). Closer evaluation of T- and FSH-driven signaling, however, reveals that the actions of these hormones affect overlapping pathways, as both FSH and T activate the MAP/ERK and CREB signaling cascades (25), recently shown to be crucial for murine spermatogenesis through a rapid T signaling mechanism (26). FSH and T also have nonadditive effects on SC intracellular levels of free Ca^2+^, possibly mediated by the same Ca^2+^ channels (27, 28). Consequently, T and FSH signaling pathways are partly overlapping, but strong FSH stimulation in the absence of T is required to observe the FSH effect on classical androgen-regulated genes. The incomplete recovery of quantitative spermatogenesis and fertility in the *Fshr*-CAM/*Lhr*^–/–^ mice emphasizes that qualitatively and quantitatively full spermatogenesis requires T.

Our finding of strong FSH stimulation maintaining spermatogenesis in the absence of T in mice is in all probability similar to what occurs in humans and could explain the equivocal effects of FSH therapy in the treatment of idiopathic oligozoospermia (29, 30). We believe that strong FSH action can boost spermatogenesis beyond that achieved at physiological levels and may even compensate for missing or insufficient T action, e.g., in oligozoospermia due to partial androgen resistance. Indeed, a study using 3- to 6-fold higher than the standard dose of FSH (75 IU, 2 to 3 times weekly) showed significant stimulation of spermatogenesis in idiopathic oligozoospermia (31). Besides increased FSH doses, the recently developed small molecule allosteric agonists of glycoprotein hormones (32) could offer a future alternative to boost FSHR activation. These findings may also explain the mechanism of persistent spermatogenesis in a hypophysectomized male with activating *FSHR* mutation (11) and suggest a role for FSH in the LH/T-deficient Pasqualini syndrome (fertile eunuch) (33). Clearly, spermatogenesis is possible without T, and the potential of strong FSH stimulation in the treatment of spermatogenic failure needs further attention. Finally, our findings provide insight into the perplexing shift in the hormonal regulation of spermatogenesis during evolution from FSH in teleost fishes to LH/T dominance in mammals (34).

## Methods

### Statistics.

Single comparisons were performed with unpaired 2-tailed Student’s *t* tests and multiple comparisons using ANOVA and Newman-Keuls post hoc test. All data sets are presented as mean ± SEM, unless otherwise stated. *P* < 0.05 was considered statistically significant.

### Study approval.

All procedures conformed to the Imperial College London Animal Welfare Protocol and were approved in accordance with the regulations and standards of the UK Home Office Animal Scientific Procedures Act (ASPA) 1986 and the European Union Directive (2010).

For additional information, see Supplemental Methods.

## Author contributions

OOO, HP, and ITH designed the study. OOO, HP, AP, LV, MC, MD, LS, LO, and BK performed experiments and collected data. OOO, HP, AP, LV, MC, MD, LS, NAR, and ITH analyzed the data. OOO and ITH drafted the manuscript, with final editing from all authors.

## Supplementary Material

Supplemental data

## Figures and Tables

**Table 1:**
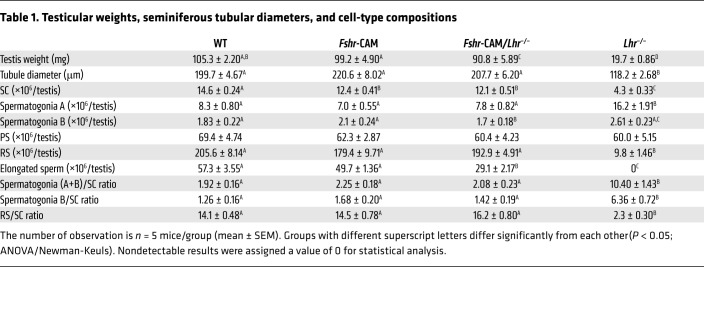
Testicular weights, seminiferous tubular diameters, and cell-type compositions

## Version 1 03/26/2018

Electronic publication

## Version 2 05/01/2018

Print issue publication
