# Supplementary material for: Constitutively active follicle-stimulating hormone receptor enables androgen-independent spermatogenesis
Source: J Clin Invest. 2018 Mar 26;128(5):1787–92. doi: 10.1172/JCI96794 (PMC5919831; doi:10.1172/JCI96794)
Supplement: Supplemental data [file jci-128-96794-s001.pdf]

Supplemental Information

Constitutively active follicle-stimulating hormone receptor enables androgen-independent spermatogenesis

Olayiwola O. Oduwole,[1§] Hellevi Peltoketo,[1,2§] Ariel Poliandri,[1,3] Laura Vengadabady,[4] Marcin Chrusciel,[5] Milena Dorozko,[5] Luna Samanta,[6] Laura Owen,[7] Brian Keevil,[7] Nafis A. Rahman,[5,8] Ilpo T. Huhtaniemi,[1,5]*

[1]Institute of Reproductive and Developmental Biology, Department of Surgery and Cancer, Imperial College London, Hammersmith Hospital Campus, London, United Kingdom.

[2]Laboratory of Cancer Genetics and Tumor Biology, Cancer and Translational Medicine Research Unit/Laboratory Medicine, Biocenter Oulu and Faculty of Medicine, University of Oulu, Oulu, Finland.

[3]Department of Molecular and Clinical Sciences, St. George's University of London, London, United Kingdom.

[4]Department of Target Sciences, GlaxoSmithKline, London, United Kingdom.

[5]Department of Physiology, University of Turku, Turku, Finland.

[6]Department of Zoology, School of Life Sciences, Ravenshaw University, Cuttack, India.

[7]Biochemistry Department, University Hospital of South Manchester, Manchester, United Kingdom.

[8]Department of Reproduction and Gynecological Endocrinology, Medical University of Bialystok, Bialystok, Poland

[§]These authors contributed equally to this work.

*Corresponding author: Ilpo T. Huhtaniemi, IRDB, Department of Surgery and Cancer, Imperial College London, Hammersmith Hospital Campus, Du Cane Road, London W12 0NN, UK. Tel: +44 (0)20 7594 2107. E-mail: ilpo.huhtaniemi@imperial.ac.uk

Methods

Reagents:

All reagents, unless otherwise stated, were purchased from Sigma Chemical Co. (St. Louis, MO).

Mouse breeding:

The detailed strategies for the generation of the transgenic mutant *Fshr*^{D580H} [FVB/N-Tg(AMH-FSHR^{D580H})/IC] (abbreviated as *Fshr*-CAM) mice (1) and *Lhr*$^{-/-}$ mice (2) were as previously described. To generate the double mutant *Fshr*-CAM mice on a homozygous *Lhr*$^{-/-}$ background (*Fshr*-CAM/*Lhr*$^{-/-}$), the *Lhr*$^{+/-}$ females were first backcrossed to FVB/N background, by intercross with the male transgenic *Fshr*-CAM mice to obtain the *Fshr*-CAM/*Lhr*$^{+/-}$ males, which were further crossbred with *Lhr*$^{+/-}$ females. Distribution of the genotypes was according to estimated Mendelian inheritance (Supplemental Table 5). All mice were identified by genotyping with specific primers (Supplemental Table 6) and were randomly assigned to different groups to minimize potential bias.

Fertility assessment:

Initial mounting behaviour of adult male *Fshr*-CAM/*Lhr*$^{-/-}$ and WT littermates was assessed through preliminary mating with WT females, aged 5-6 weeks, stimulated by the standard superovulation technique. Each superovulated female was paired overnight with a male under assessment. Vaginal plugs indicating successful mating were checked early the following morning. For fertility assessment, *Fshr*-CAM/*Lhr*$^{-/-}$ and WT male mice aged 6-7 weeks (n=3/group) were paired with randomly selected WT females aged 5-6 weeks for continuous breeding, to assess pregnancy rates and litter sizes.

Antiandrogen treatment:

7.5-mg 90-day slow-release nonsteroidal antiandrogen flutamide pellets (Innovative Research of America, Sarasota, FL) were implanted subcutaneously under isoflurane anesthesia into 2-month old *Fshr*-CAM/*Lhr*$^{-/-}$ mice and age-matched WT littermates (n=5/group). Equal number of mice (n=5/group) from each group were treated with placebo implants and served as controls. At the end of the experiment, reproductive and accessory sex organs were collected, weighed and visualized for changes. Morphology of the testes was assessed by hematoxylin and eosin (H & E) staining.

Sample collection and preparation:

Mice were anesthetized with 2-2-2-tribromoethanol by intraperitoneal injection (3) and exsanguinated by cardiac puncture followed by cervical dislocation. Serum samples were separated by centrifugation and stored at –20 °C. Right testes were stored at –80 °C for iTT measurement and gene expression analyses. The left testes were processed for histology, immunohistochemistry (IHC), and RNAscope *in situ* hybridization (ISH).

Hormone measurements:

LH and FSH concentrations were measured by immunofluorometric assays (4, 5). Serum T and iTT were measured with Waters Acquity UPLC and Waters TQS tandem mass spectrometer (Waters, Manchester, UK) (6). For iTT measurement, frozen right testes were weighed, decapsulated and homogenized in PBS. Debris were removed by centrifugation and supernatant used for T measurements (7, 8). The lower limit of quantitation for T and iTT were 0.1 nmol/L with an assay imprecision CV of <9%.

Histology and immunohistochemistry:

Tissues were fixed in either Bouin's solution for the histology of general tissue structures and stereology, or 10% neutral buffered formalin solution for IHC and RNAscope ISH. Fixation was at 4 °C overnight, after which tissues were dehydrated in 2-3 changes each of graduated ethanol solutions until absolute water-free ethanol, cleared in histoclear (National Diagnostics, Hessle Hull, UK) and embedded in paraffin wax. Cut serial sections of 5 µm thickness were mounted on polylysine microscope slides (VWR, Lutterworth, UK), dried at 37 °C for 1 hour, and stored until required. Sections for histology were stained with the standard H&E protocol, viewed with Nikon Eclipse ME600 microscope and photomicrographs taken with a mounted Nikon D1500 digital camera.

For the IHC of SF1, StAR, HSD3B1, CYP17A1 and HSD17B3, sections were deparaffinized in xylene, washed in decreasing concentration of ethanol solutions and several changes of water. Antigen retrieval was with 10 mM citrate buffer (pH 6.0) using the 2100 Retriever (Aptum Biologics Ltd., Southampton, UK). Non-specific binding was blocked with 3% bovine serum albumin (BSA). Incubation with specific primary antibodies (Supplemental Table 7) was carried out overnight at 4 °C. Thereafter, sections were washed in Tris-Buffered Saline (TBS) containing 0.1% Tween-20 (TBS-T), followed by incubation in Dako EnVision^{+} System-HRP (DAB) polymer anti-mouse (K4007, Dako, Glostrup, Denmark), or anti-rabbit (K4011, Dako) secondary antibody and visualized using Bright-DAB reagent (IL Immunologic, Duiven, Holland). Counterstaining was with Mayer's hematoxylin (Histolab Products AB, Gothenburg, Sweden). Sections were dehydrated in ethanol solutions, cleared in xylene and mounted with Pertex (Histolab Products AB). Slides were scanned with Pannoramic 250 Slide Scanner (3D HISTECH Ltd., Budapest, Hungary) and pictures taken using Pannoramic Viewer (3D HISTECH).

The procedure for FSHR staining was modified from Radu et al (9). After antigen retrieval, endogenous peroxidase activity was blocked with 6% hydrogen peroxide solution at room temperature (RT). Sodium borohydride (10 mg/mL in PBS) was used to quench free aldehyde groups, while non-specific binding of antibody was blocked with 3% BSA in PBS containing 0.05% Tween 20 (PBS-T) at RT. Primary antibodies were linked with Envision® anti-mouse polymer conjugated to horseradish peroxidase (HRP) enzyme (#K4007, Dako). The reaction product was visualized using 3,3'-diaminobenzidine tetrahydrochloride (DAB, Dako). Sections were counterstained with Mayer's hematoxylin (Histolab Products AB), dehydrated in grades of ethanol and mounted with Pertex (Histolab Products AB). Photomicrographs were captured with Zeiss AxioImager M1 and ZEN 2 (blue edition) software (Carl Zeiss GmbH, Vienna, Austria).

RNAscope *in situ* hybridization (ISH):

Testicular sections of 5 µm thickness (n=3/group) were subjected to ISH using RNAscope® 2.5 HD Reagent Kit-BROWN (Advanced Cell Diagnostics, Newark, CA) according to manufacturer's protocol and as previously described (10). Hybridization with probes against mouse Fshr (#400461), Polr2a (positive control, #312471) or dapB (negative control, #310043) was performed in HybEZ™ Oven (Advanced Cell Diagnostics). Sections were scanned using Pannoramic Midi FL slide scanner (3DHISTECH Ltd.) and pictures taken using Pannoramic Viewer (3DHISTECH).

Stereological assessment:

Photomicrographs of the H & E stained slides were captured with the NanoZoomer 2.0-HT digital slide scanner, analyzed with the NDPview software (Hamamatsu Photonics, Welwyn Garden City, UK). Five slides of serial sections from the middle of each testis were examined from each group (n=5/group). Three sections from each slide were selected at random and

analyzed for cell-type composition. Quantitative analyses of spermatogenesis were carried out by counting the number of Sertoli cells, types A and B spermatogonia, and pachytene spermatocytes and round spermatids, steps 1 to 8 (11). Nuclei of different germ cells were counted in 100 round seminiferous tubule cross sections. Counts were subjected to a modified Abercrombie correction for section thickness and differences in the nuclear or nucleolar diameter (12, 13). The total number of germ cells per testis was determined as: Numerical volume (N_V) x Reference volume (R_V) of the testis; where $N_V = N_A/ (T + D – 2h)$, N_A = number of nuclei per unit area, T = section thickness, D = nuclear diameter, and h = height of the smallest visible cap section (h is usually 1/10th of nuclear diameter of the cell type counted, for example in pachytene spermatocytes usually 0.5 µm). R_V is the weight of the testis/density of the testis per mL (average density of testis = 1.04 g/mL) (14, 15). The numerical densities of elongated spermatids (steps 15–19) per testis were counted from the photomicrographs of lumens from three randomly selected testicular cross-sections, and the average of the total counts taken to obtain a mean count for each individual animal (16).

Quantitative Real-Time RT-PCR:

Total RNA (tRNA) was purified from testicular tissues with TriReagent. 1 µg of tRNA was reverse-transcribed to cDNA using SuperScript II First Strand Synthesis Kit (Invitrogen, Carlsbad, CA). Quantitative PCR (qPCR) was performed on diluted cDNA samples using SYBR Green JumpStart Taq ReadyMix reagent in an ABI Step One Plus QPCR system (Applied Biosystems, Carlsbad, CA) with gene-specific primers sequences (Supplemental Table 6), using the program: 95 °C for 10 min, 40 cycles at 95 °C for 15 seconds, 60 °C for 30 seconds, and 72 °C for 30 seconds. A minimum of three mice per genotype were analyzed, and each gene was analyzed three times in duplicates per assay. The mean results of all three

assays were analyzed using the $2^{-\Delta\Delta Ct}$ method (17). Expression of all genes was normalized against the geometric mean of ribosomal protein L19 (*RpL19)* and beta actin (*Actb*).

Supplemental References

1. Peltoketo H, et al. Female mice expressing constitutively active mutants of FSH receptor present with a phenotype of premature follicle depletion and estrogen excess. *Endocrinology.* 2010;151(4):1872-1883.
2. Zhang FP, Poutanen M, Wilbertz J, Huhtaniemi I. Normal prenatal but arrested postnatal sexual development of luteinizing hormone receptor knockout (LuRKO) mice. *Mol Endocrinol.* 2001;15(1):172-183.
3. Weiss J, Zimmermann F. Tribromoethanol (Avertin) as an anaesthetic in mice. *Lab Anim.* 1999;33(2):192-193.
4. Haavisto AM, Pettersson K, Bergendahl M, Perheentupa A, Roser JF, Huhtaniemi I. A supersensitive immunofluorometric assay for rat luteinizing hormone. *Endocrinology.* 1993;132(4):1687-1691.
5. van Casteren JI, Schoonen WG, Kloosterboer HJ. Development of time-resolved immunofluorometric assays for rat follicle-stimulating hormone and luteinizing hormone and application on sera of cycling rats. *Biol Reprod.* 2000;62(4):886-894.
6. Owen LJ, Keevil BG. Testosterone measurement by liquid chromatography tandem mass spectrometry: the importance of internal standard choice. *Ann Clin Biochem.* 2012;49(Pt 6):600-602.
7. Shetty G, Wilson G, Huhtaniemi I, Shuttlesworth GA, Reissmann T, Meistrich ML. Gonadotropin-releasing hormone analogs stimulate and testosterone inhibits the recovery of spermatogenesis in irradiated rats. *Endocrinology.* 2000;141(5):1735-1745.

8. Oduwole OO, et al. Overlapping dose responses of spermatogenic and extragonadal testosterone actions jeopardize the principle of hormonal male contraception. *FASEB J.* 2014;28(6):2566-2576.

9. Radu A, et al. Expression of follicle-stimulating hormone receptor in tumor blood vessels. *N Engl J Med.* 2010;363(17):1621-1630.

10. Wang F, et al. RNAscope: a novel in situ RNA analysis platform for formalin-fixed, paraffin-embedded tissues. *J Mol Diagn.* 2012;14(1):22-29.

11. Russell LD, Clermont Y. Degeneration of germ cells in normal, hypophysectomized and hormone treated hypophysectomized rats. *Anat Rec.* 1977;187(3):347-366.

12. Abercrombie M. Estimation of nuclear population from microtome sections. *Anat Rec.* 1946;94:239-247.

13. Amann RP. Reproductive capacity of dairy bulls. IV. Spermatogenesis and testicular germ cell degeneration. *Am J Anat.* 1962;110:69-78.

14. Mori H, Christensen AK. Morphometric analysis of Leydig cells in the normal rat testis. *J Cell Biol.* 1980;84(2):340-354.

15. Yang ZW, Wreford NG, de Kretser DM. A quantitative study of spermatogenesis in the developing rat testis. *Biol Reprod.* 1990;43(4):629-635.

16. Wing TY, Christensen AK. Morphometric studies on rat seminiferous tubules. *Am J Anat.* 1982;165(1):13-25.

17. Livak KJ, Schmittgen TD. Analysis of relative gene expression data using real-time quantitative PCR and the 2(-Delta Delta C(T)) Method. *Methods.* 2001;25(4):402-8.

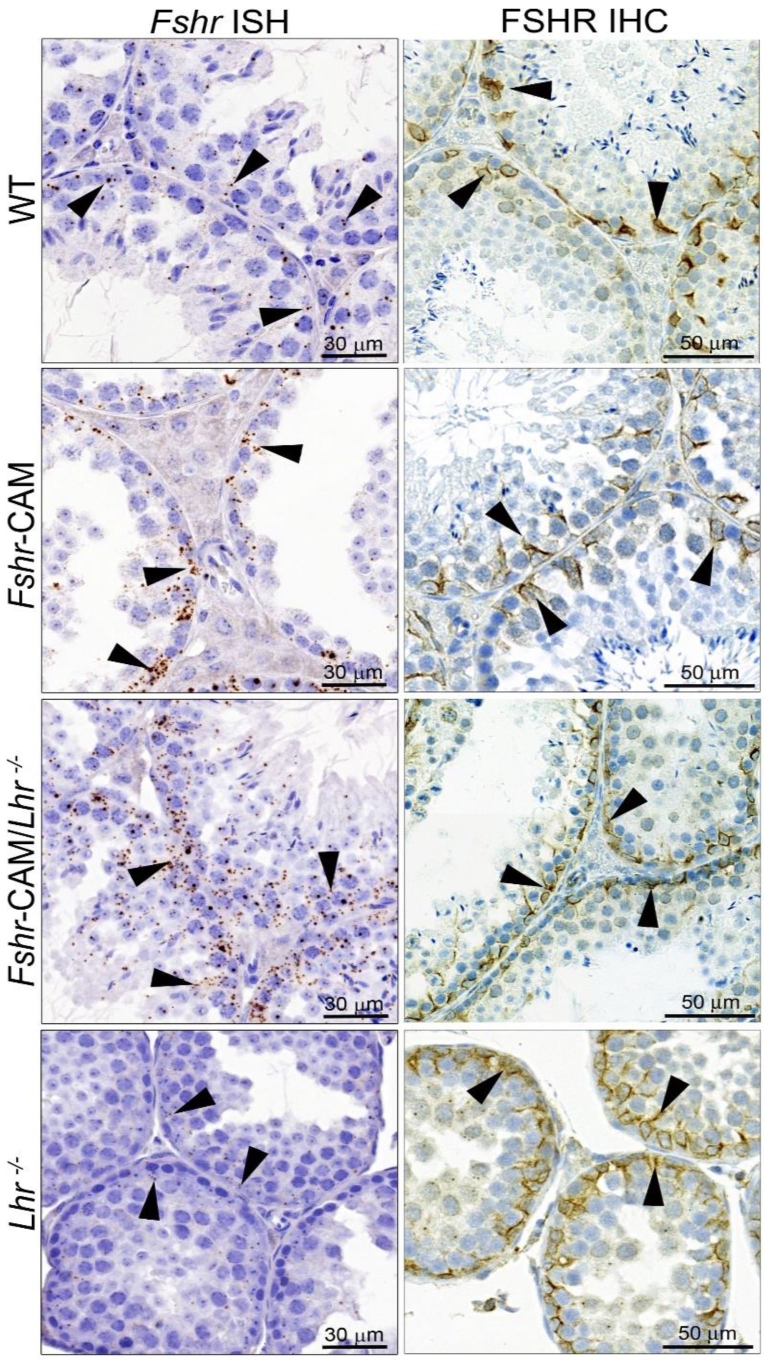

Supplemental Figure 1. ***In situ*** **hybridization (ISH, left panels) and immunohistochemical (IHC, right panels) localization of FSHR mRNA and protein, respectively, in the testes of** WT, *Fshr*-CAM, *Fshr*-CAM/*Lhr*$^{-/-}$ and *Lhr*$^{-/-}$ mice. These data show the expression of FSHR at mRNA and protein levels in all phenotypes confined exclusively to SC (arrow heads). They also confirm the SC specificity of the *Amh*-promoter driven *Fshr*-CAM transgene and imply that the slightly elevated serum T observed in the *Fshr*-CAM/*Lhr*$^{-/-}$ mice, as compared to the *Lhr*$^{-/-}$mice, was not a result of leaky *Fshr*-CAM expression into LC. Representative images of $\geq$3 samples per treatment group are presented.

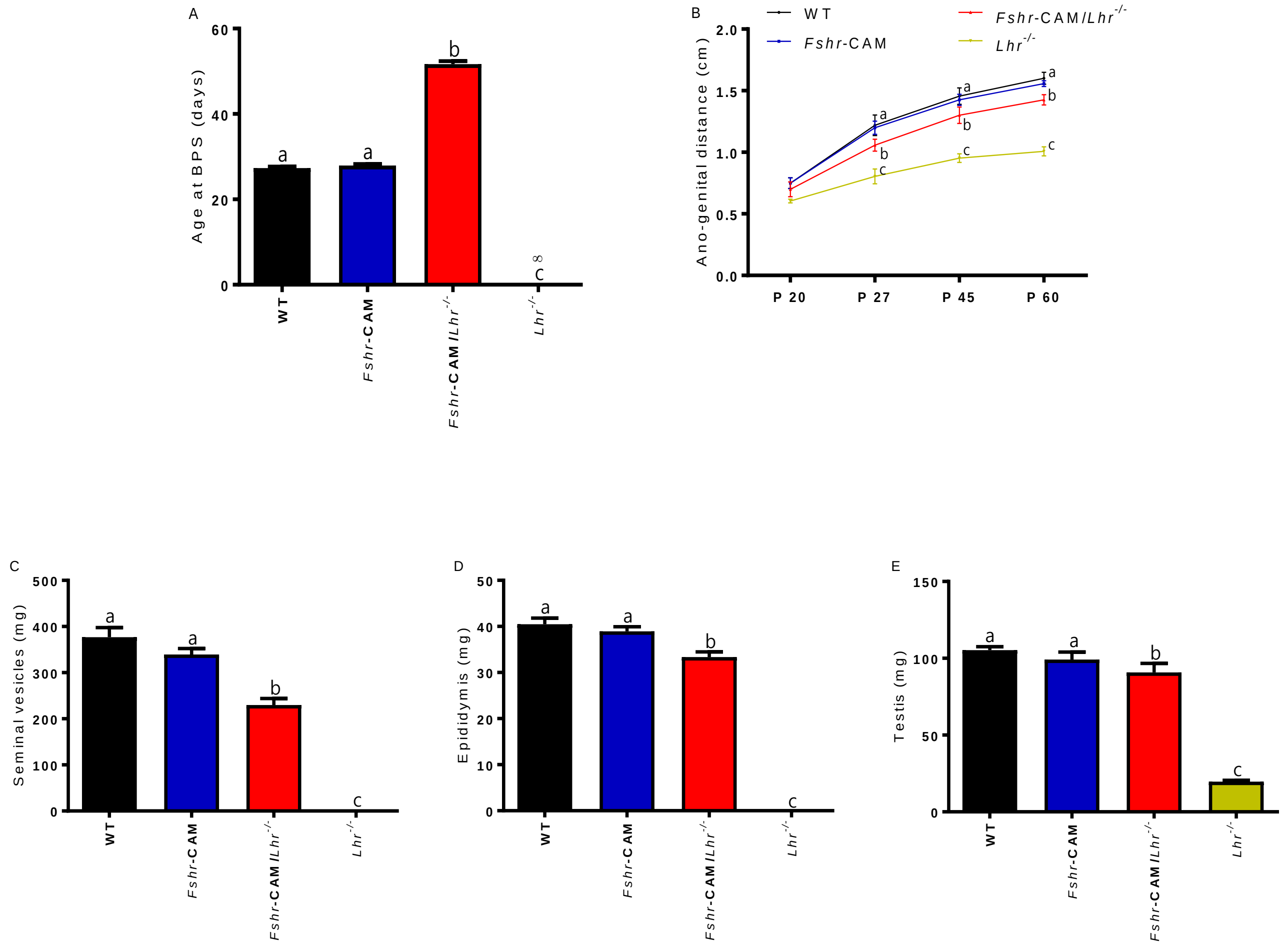

Supplemental Figure 2. (**A**) Age at balano-preputial separation. (**B**) Anogenital distance between days P20-P60. (**C**) Weights of seminal vesicles. (**D**) Weights of epididymides. (**E**) Weights of testes. All samples were from 3-month-old WT, *Fshr*-CAM, *Fshr*-CAM/*Lhr*$^{-/-}$ and *Lhr*$^{-/-}$ mice. Data are mean ± SEM, n = 10-15 mice/group. Bars with different superscript letters differ significantly from each other (P at least <0.05; ANOVA/Newman-Keuls).

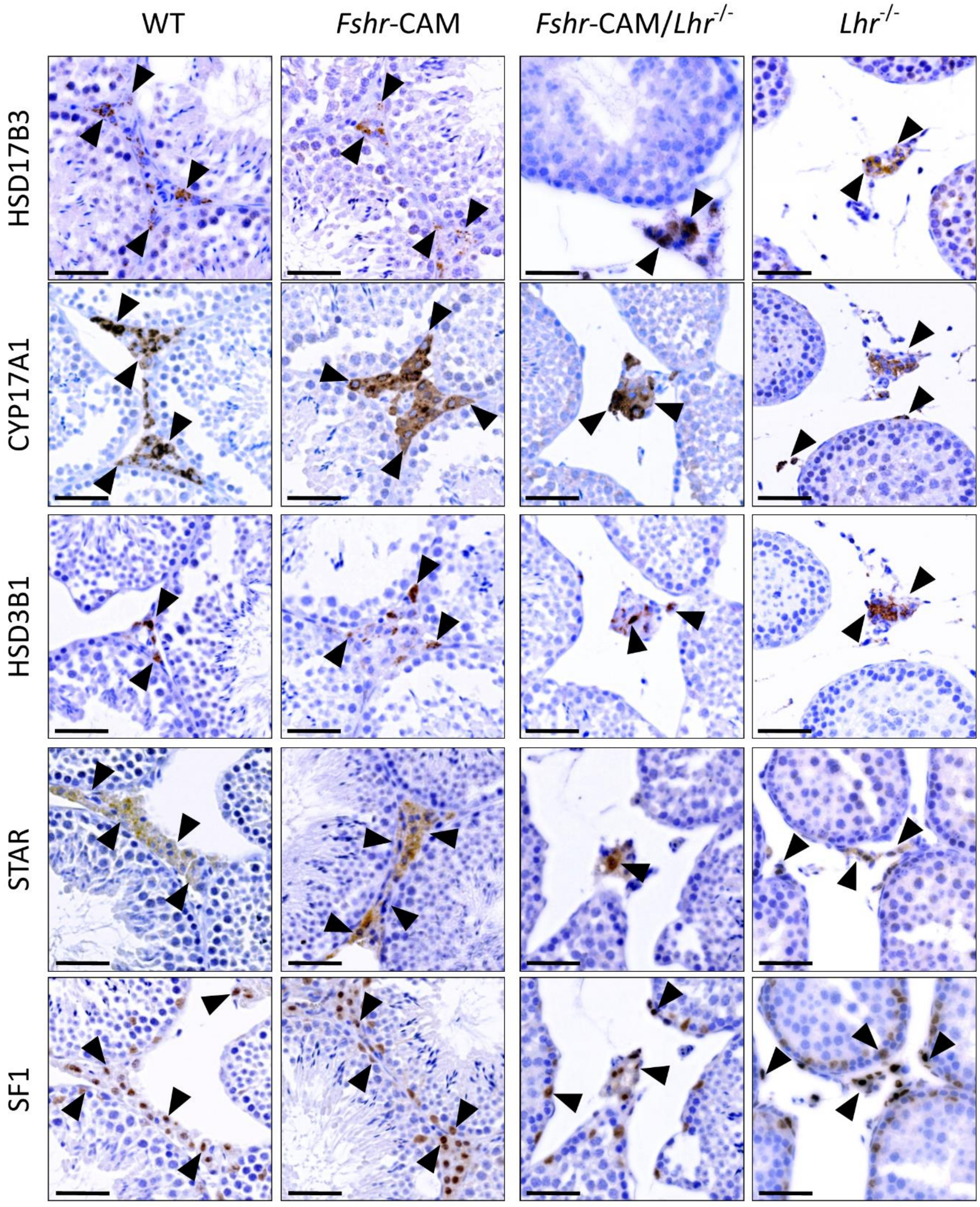

Supplemental Figure 3. Immunohistochemical localization of steroidogenic enzymes HSD17B3, CYP17A1, HSD3B1, transport protein StAR and transcription factor SF1. These data show StAR and the enzymes of androgen biosynthesis exclusively expressed in LC (arrow heads), while SF1 is expressed in both LC and SC of the transgenic mice like in WT mice. Bar = 50 μm. Representative images of $\geq$3 samples per treatment group are presented.

	WT	*Fshr*-CAM
Testis weight (mg)	99.5 ± 11.9	106 ± 19.3
LH (µg/L)	0.25 ± 0.16	0.32 ± 0.36
FSH (µg/L)	40.0 ± 7.3	44.2 ± 7.5
T (nmol/L)	7.7 ± 13.0	6.2 ± 11.4

Supplemental Table 1. Testis weight, and hormonal data in WT and *Fshr*-CAM mice. Testis weights and serum concentrations of LH, FSH and T in groups of WT (n=29) and *Fshr*-CAM (n=38) mice. Data are presented as mean ± SD. No significant differences were found between the groups in any of the parameters (Student's *t* test).

Genotype	Number of litters	Total number of pups	Average number of pups per litter
WT	22	196	8.9 ± 0.28
Fshr-CAM/*Lhr*$^{-/-}$	5	17	3.4 ± 0.39*

Supplemental Table 2. Reproductive performance in WT and *Fshr*-CAM/*Lhr*$^{-/-}$ mice. Data from three breeding pairs of a male with a wild-type female mouse were evaluated (n=3/group). *indicates significant difference from WT ($P < 0.05$; Student's *t* test). Although the *Fshr*-CAM/*Lhr*$^{-/-}$ were able to sire offspring, their fertility was only partially recovered, likely due in part to the insufficient T levels of these animals.

	WT	*Fshr*-CAM	*Fshr*-CAM/*Lhr*$^{-/-}$	*Lhr*$^{-/-}$
SC: x10^6/mg testis	0.14 ± 0.004^a	0.12 ± 0.004^b	0.13 ± 0.005^b	0.22 ± 0.02^c
Spermatogonia A: $^x10^6$/mg testis	0.08 ± 0.007^a	0.07 ± 0.005^a	0.08 ± 0.008^a	0.82 ± 0.09^b
Spermatogonia B: $_x10^6$/mg testis	0.02 ± 0.002^a	0.02 ± 0.002^a	0.02 ± 0.002^a	0.13 ± 0.01^b
PS: x10^6/mg testis	0.66 ± 0.045^a	0.63 ± 0.03^a	0.66 ± 0.046^a	3.04 ± 0.26^b
RS: x10^6/mg testis	1.95 ± 0.08^a	1.80 ± 0.01^a	2.12 ± 0.054^a	0.49 ± 0.16^b
Elongated sperm: $^x10^6$/mg testis	0.54 ± 0.033^a	0.50 ± 0.001^a	0.32 ± 0.024^b	0^c
Spermatogonia (A+B)/SC ratio	1.83 ± 0.18^a	2.25 ± 0.17^a	2.08 ± 0.23^a	10.40 ± 1.43^b
Spermatogonia B/SC ratio	1.26 ± 0.16^a	1.68 ± 0.20^a	1.42 ± 0.19^a	6.36 ± 0.72^b
RS/SC ratio	14.1 ± 0.48^a	14.5 ± 0.78^a	16.2 ± 0.80^a	2.3 ± 0.30^b

Supplemental Table 3. Testicular cell-type composition analysis in mg per testis. Further details are presented in Table 1 (main manuscript). Number of observation is n=5 mice/group (mean ± SEM). Different superscript letters indicate significant differences from each other ($P < 0.05$; ANOVA/Newman-Keuls). Non-detectable results were assigned a value of 0 for statistical analysis.

	WT Placebo	WT Flutamide	*Fshr*-CAM/*Lhr*$^{-/-}$ Placebo	*Fshr*-CAM/*Lhr*$^{-/-}$ Flutamide
Testis weight (mg)	104.9 ± 1.76^{a}	21.9 ± 0.46^{b}	91.06 ± 1.30^{c}	88.68 ± 1.16^{c}
Tubule diameter (μm)	198.8 ± 6.68^{a}	137.0 ± 4.28^{b}	201.2 ± 2.36^{a}	198.2 ± 1.86^{a}
SC: x10^{6}/testis	14.2 ± 0.32^{a}	5.4 ± 0.67^{b}	12.0 ± 0.29^{c}	11.9 ± 0.20^{c}
Spermatogonia A: x10^{6}/testis	8.3 ± 0.53^{a}	14.2 ± 0.76^{b}	7.0 ± 0.37^{a}	6.6 ± 0.29^{a}
Spermatogonia B: x10^{6}/testis	1.8 ± 0.28	2.6 ± 0.29	1.7 ± 0.29	1.6 ± 0.24
PS: x10^{6}/testis	70.3 ± 3.64	61.7 ± 3.92	61.9 ± 2.49	59.8 ± 1.49
RS: x10^{6}/testis	201.3 ± 4.31^{a}	10.8 ± 1.61^{b}	190.2 ± 2.82^{c}	186.1 ± 3.77^{c}
Elongated sperm ^{x}10^{6}/testis	57.8 ± 5.10^{a}	0^{b}	28.6 ± 0.96^{c}	26.7 ± 1.28^{c}

Supplemental Table 4. Testicular cell-type composition analysis of flutamide and placebo treated WT and *Fshr*-CAM/*Lhr*$^{-/-}$ mice. Number of observation is n=5 mice/group (mean ± SEM). Different superscript letters indicate significant differences (P at least < 0.05; ANOVA/Newman-Keuls). Non-detectable results were assigned a value of 0 for statistical analysis.

	Males	Females	Total	Observed (%)	Expected (%)
WT	44	53	97	12.7	12.5
WT/*Lhr*$^{+/-}$	105	109	214	28	25
WT/*Lhr*$^{-/-}$	46	40	86	11.2	12.5
Sum	195	202	397	51.9	50
Fshr-CAM/WT	39	35	74	9.7	12.5
Fshr-CAM/*Lhr*$^{+/-}$	98	107	205	26.8	25
Fshr-CAM/*Lhr*$^{-/-}$	42	47	89	11.6	12.5
Sum	179	189	368	48.1	50
Total of sums	253	286	765	100	100

Supplemental Table 5. Distribution of genotypes. Distribution of the different genotypes in males and females with observed and expected percentage of total shown.

Supplemental Table 6. Primer sequences

Gene	Accession No.		Primer sequence	Reference or Figure No.
Standard genotyping PCR primers				
m/rFSHR1			AGT TCA ATG GCG TTC CGG	Ref. 8/
AMH-prom3			ACG GCA TGT TGA CAC ATC AG	Suppl. Ref. 1
mFSHR10			TGA TTA TCT CAA CAG AGT TGG	
mFSHR11			GTG GTT GAG CCT ATA CAT GG	
LHR1			TCT GGG GAT CTT GGA AAT GA	Ref. 9/
LHR2			CAC CTT GAC ACC TGG AGT	Suppl. Ref. 2
Neo A			GGG CTC TAT GGC TTC TGA GGC GGA	
qPCR primers				
Scd1	NM_009127.4	FW	AGG CGA GCA ACT GAC TAT CA	Figure 3A
		RV	CCG TCT TCA CCT TCT CTC GT	
Cyp11a1	NM_019779.3	FW	CAC TCC TCA AAG CCA GCA TC	Figure 3A
		RV	GCA AAG CTA GCC ACC TGT AC	
Cyp17a1	NM_007809.3	FW	CGT CTT TCA ATG ACC GGA CT	Figure 3A
		RV	CAT AAA CCG ATC TGG CTG GT	
Star	NM_011485.4	FW	ACT CAC TTG GCT GCT CAG TA	Figure 3A
		RV	AGT CCT TAA CAC TGG GCC TC	
Hsd17b3	NM_008291.3	FW	CGG GAA AGC CTA TTC ATT TG	Figure 3A
		RV	TCA CAC AGC TTC CAG TGG TC	
Cyp19a1	NM_007810.3	FW	GCA CAG GCT CGA GTA CTT CC	Figure 3A
		RV	CAA AGC CAA AAG GCT GAA AG	
Igf-1	NM_001111276.1	FW	TGG ATG CTC TTC AGT TCG TG	Figure 3B
		RV	GCA ACA CTC ATC CAC AAT GC	
Igf-bp3	NM_008343.2	FW	AAG TTC CAT CCA CTC CAT GC	Figure 3B
		RV	CCT CTG GGA CTC AGC ACA TT	
Amh	NM_007445.2	FW	GGG AGA CTG GAG AAC AGC AG	Figure 3B
		RV	GTC CAC GGT TAG CAC CAA AT	
Lep	NM_008493.3	FW	CCA GGA TGA CAC CAA AAC CC	Figure 3B
		RV	TGA AGT CCA AGC CAG TGA CC	

Inh 1	NM_010564.4	FW	CTC CCA GGC TAT CCT TTT CC	Figure 3B
		RV	GGA TGG CCG GAA TAC ATA AG	
Inh 2	NM_008381.3	FW	CGA GAT CAT CAG CTT TGC AG	Figure 3B
		RV	GGG GAG CAG TTT CAG GTA CA	
InsL3	NM_013564.7	FW	CAC GCA GCC TGT GGA GAC	Figure 3B
		RV	CTG AGA AGC CTG GAG AGG AA	
Esr1	NM_019437.3	FW	TCT CTG GGC GAC ATT CTT CT	Figure 3C
		RV	GCT TTG GTG TGA AGG GTC AT	
Esr2	NM_207707.1	FW	GAA GCT GGC TGA CAA GGA AC	Figure 3C
		RV	AAC GAG GTC TGG AGC AAA GA	
Ar	NM_013476.3	FW	GGA CCA TGT TTT ACC CAT CG	Figure 3C
		RV	TCG TTT CTG CTG GCA CAT AG	
Prlr	NM_001163530.1	FW	GGA TCA TTG TGG CCG TTC TC	Figure 3C
		RV	CGG AAC TGG TGG AAA GAT GC	
Lhcgr	NM_013582.2	FW	CTG AAA ACT CTG CCC TCC AG	Figure 3C
		RV	AAT CGT AAT CCC AGC CAC TG	
Fshr	NM_013523.3	FW	TGA TGT TTT CCA GGG AGC CT	Figure 3C
		RV	TAT GTT GAC CTG GCC CTC AA	
Drd4	NP_001139357.1	FW	CGT CTC TGT GAC ACG CTC AT	Figure 3 D and E
		RV	CAC TGA CCC TGC TGG TTG TA	
Gata 1	NM_008089.2	FW	AGC ATC AGC ACT GGC CTA CT	Figure 3 D and E
		RV	AGG CCC AGC TAG CAT AAG GT	
Rhox 5	NM_008818	FW	AAT GGA AAT CCT GGG GGT AG	Figure 3 D and E
		RV	CAC ACA GGC ATC CAT CAG TC	
Aqp8	NM_001109045.1	FW	TTG CTA CCT TGG GGA ACA TC	Figure 3 D and E
		RV	CCA AAT AGC TGG GAG ATC CA	
Eppin	NM_029325.2	FW	GGC TGC CAA GGA AAC AAT AA	Figure 3 D and E
		RV	TGG AGC AGA AGC CAA ATT CT	

Tjp1	NM_009386	FW	GTC TGC CAT TAC ACG GTC CT	Figure 3 D and E
		RV	TGG AGA TGA GGC TTC TGC TT	

Target protein	Manufacturer	Catalogue number	Dilution
FSHR323	Gift from Dr. Nicola Ghinea	See Ref. 9	2.5 µg/µl
HSD17B3	Proteintech Group	#13415-1-AP	1:100
CYP17A1	Proteintech Group	#14447-1-AP	1:7000
HSD3B2	Sigma-Aldrich	#SAB1402232	1:1600
STAR (D10H12) XP®	Cell Signal Technology	#8449	1:50
STF-1 (D1Z2A) XP®	Cell Signal Technology	#12800	1:50

Supplemental Table 7. List of primary antibodies used for IHC and RNAscope ISH.
